# Echocardiographic characteristics of primary malignant pericardial mesothelioma and outcomes analysis: a retrospective study

**DOI:** 10.1186/s12947-018-0125-z

**Published:** 2018-04-26

**Authors:** Lingyun Kong, Ziwang Li, Jingrui Wang, Xiuzhang Lv

**Affiliations:** 1 Echocardiography Department of Heart Center, Beijing Chao-Yang Hospital, Capital Medical University, 8 Gongren Tiyuchang Nanlu, Chaoyang District, Beijing, 100020 China; 2 Department of Cardiology, Jiang Xi Yichun Hospital of Traditional Chinese Medicine, Jiang Xi, China; 3 Department of Cardiology, Beijing Daxing District people’s Hospital, Beijing, China

**Keywords:** Pericardial mesothelioma, Echocardiography, Diagnosis, Prognosis

## Abstract

**Background:**

Little is known about the echocardiographic characteristics of primary malignant pericardial mesothelioma (PPM) due to its rarity. The aim of this study was to explore the sex-specific echocardiographic patterns of PPM and risk factors for in-hospital mortality.

**Methods:**

A retrospective information retrieval was conducted for cases of PPM reported from China during 1981 and 2015. The diagnosis was made by histopathological examinations and only cases with echocardiographic descriptions were included. Data on the clinical and echocardiographic findings were collected. Difference in clinical, sex-specific echocardiographic characteristics and findings across different time periods were assessed. Logistic regression analysis was performed to explore echocardiographic risk factors for in-hospital mortality.

**Results:**

A total of 64 patients with PPM were included, with a mean age of 39.2 ± 15.6 years and minor male dominance (40, 62.5%). The most common echocardiographic presentations were pericardial effusion (55, 85.9%), pericardial masses (36.4%) and thickening (17.3%), respectively. The positive rate of pericardiocentesis was only 20.9%. Six patients (15.4%) died among 39 cases reporting in-hospital outcome. Logistics analysis identified no clinical or echocardiographic parameters associated with in-hospital mortality (all *P* > 0.05).

**Conclusions:**

The echocardiographic signs of PPM are basically nonspecific with massive pericardial effusion as the most common sign, although no echocardiographic gender differences or association with in-hospital mortality could be identified.

## Background

Primary malignant pericardial mesothelioma (PPM) is an extremely rare malignancy originating from the pericardium, with an incidence < 0.0022% [1, 2]. Little is known about the echocardiographic characteristics of PPM due to its rarity. This study was aimed to investigate the echocardiographic patterns of PPM, explore potential gender difference, the changes with time periods and echocardiographic risk factors for in-hospital mortality based on literature review of histologically proven PPM cases reported in China mainland.

## Methods

### Data sources and study population

The Chinese medical literature database including Wan Fang database, VIP database and China National Knowledge Infrastructure (CNKI) database as well as PubMed database were searched for cases of histopathologically diagnosed PPM reported from China mainland between January 1981 and December 2015. We used “pericardial mesothelioma”, “heart and mesothelioma” and “mesothelioma” as the keywords. References from the identified case reports were also reviewed. The diagnosis was based on detection of malignant mesothelial cells upon histopathological examinations including exploratory thoracotomy, biopsy and autopsy. Cases with extractable individual clinical and echocardiographic data were included. The cases may be reported in the form of case report, series or conference papers. Exclusion criteria included metastatic pericardial tumors, primary pericardial tumor of other pathological origin, repeat reports and cases diagnosed with pericardial effusion cytological tests. Particular attention was paid to cases from the same author or institution to rule out repeat cases, but patient information was extracted from all relevant publications to supplement data.

### Clinical and echocardiographic parameters

We collected clinical data including age, gender, blood pressure and heart rate. Echocardiographic data were obtained from the publications and the images, where available, provided in the literature. The signs collected included: presence, amount and color of pericardial effusion, presence, location and echodensity of pericardial mass, presence of pericardial wall thickening. The size of pericardial effusion was recorded mainly according to the reports, and the largest amount was extracted for analysis in patients having more than one echocardiogram. In the cases not describing the amount straightforward, we defined it as large ≥ 2 of the following signs were present: swimming heart, the amount > 500 mL at one pericardiocentesis/ > 1000 mL at two, or flask-like cardiomegaly on chest radiograph. The color of effusion was confirmed by both pericardiocentesis and surgery or autopsy. The frequency of pericardial mass and pericardial thickening was recorded from both echocardiography and other examinations: imaging tests like cardiac computed tomography (CT), magnetic resonance (CMR) and Positron Emission Tomography–Computed Tomography (PET-CT), and invasive exploratory thoracotomy and autopsy. The myocardial infiltration and hemodynamic complications including cardiac tamponade and constrictive pericarditis were also recorded.

The anatomical classification of PPM, including diffuse and localized type, was established according to the scale of pericardial wall involvement and presence of pericardial mass. Patients with diffuse pericardial wall thickening as evidenced by surgery, autopsy, CT, CMR or PET-CT were grouped into the diffuse type whether the mass was present or not. Patients without pericardial mass, whether there was pericardial effusion or not, were also considered as the diffuse type. Cases reported as having one or more masses in the pericardial cavity and no evidence of diffuse pericardial wall thickening were grouped into the localized type. The pathological classification was based on histopathological examinations, including epithelioid, sarcomatous and mixed (biphasic) type. Cases with pathological descriptions of nestle-like, gland-like or papillary arrangement of malignant mesothelial cells were considered as the epithelioid type.

The outcome information was retrieved when available. The primary endpoint was defined as in-hospital mortality. Two reviewers were responsible for collecting the data using the same data abstraction form. For judgment in doubt, discussion and consensus were obtained for analysis.

### Statistical analyses

Continuous data are expressed as mean ± SD, and categorical data as frequency (percentage, %). Patients were grouped according to sex to explore sex-specific difference. Considering that the study covered a relatively long range of time (35 years), during which echocardiography has undergone marked technical progress, the patients were split into two groups according to publication years: from 1981 to 2000 and from 2001 to 2015. Continuous data were compared using Student’s *t* test and categorical data were compared using χ^2^ or Fisher’s exact test. For patients with both echocardiography and other examinations, the ability of detecting pericardial thickening and mass were compared using exact McNemar test. Agreement between the echocardiography and other examinations in detecting the two signs was assessed using simple kappa (κ). Binary logistic regression analysis was used to identify risk factors for in-hospital mortality. The results were reported as Odds ratio (OR) and 95% confidence interval (CI). Analyses were performed with IBM SPSS 23.0. *P* < 0.05 was considered statistically significant.

## Results

### Retrieval information and patients demographics

**Fig. 1:**
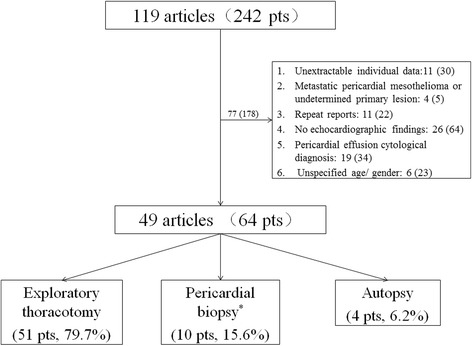
^*^One article may contain multiple cases, thus, the sum of the patients is > 119; One patient was diagnosed with two approaches, making the sum of diagnostic tests > 64. pts., patients

A total of 119 articles containing 242 PPM patients were retrieved. After exclusion of 77 articles (178 patients) not meeting the inclusion criteria: individual data unavailable 11 articles (30 patients); metastatic pericardial mesothelioma or unclear primary lesion 4 articles (5 patients); repeat reports 11 articles (22 patients); unavailable echocardiographic data 26 articles (64 patients); cytological diagnosis 19 articles (34 patients) and unspecified age or gender 6 articles (23 patients), finally 49 articles (64 patients) were enrolled (Fig. 1), among whom, 51 (79.7%, containing one case diagnosed also by biopsy) patients were diagnosed with exploratory thoracotomy, 10 (15.6%) with biopsy and 4 (6.2%) with autopsy. The clinical features and inter-gender difference of the study population is presented in Table 1. The study revealed a male/female ratio of 1.7:1 in PPM, with an increasing trend for female prevalence, which was 18.2% during 1981 and 2000, and rising to 47.6% during 2001 and 2015. The mean age was 39.5 ± 15.5 years, covering a wide range from 2 to 77 years. Notably, the age group from 19~ 65 years was mostly affected (55, 85.9%), with 7 (10.9%) patients in the 2~ 18 years group and 2 (3.1%) in the > 65 years group. There was no significant sex difference in clinical characteristics except for a lower systolic blood pressure in female patients (*P* = 0.03). In 58 patients whose morphological phenotype could be determined, the diffuse type was more common than the localized type (69.0% vs.31.0%, *P* < 0.05). 28 cases provided pathologic diagnosis or detailed description, of which the epithelioid type was most frequently found (15, 53.6%), followed by the mixed (9, 32.1%) and sarcomatous type (4, 14.3%). Pleural effusion was found in 33 (86.8%) of 38 patients, affecting mostly bilateral pleural cavity. One case reported initial onset as systematic erythroderma.

**Table 1:** Clinical characteristics of patients with PPM

Parameters	All (*n* = 64)	Group based on sex			Group based on publishing year		
		Male (*n* = 40)	Female (*n* = 24)	*P*	1981 to 2000 (*n* = 22)	2001 to 2015 (*n* = 42)	*P*
Female	24 (37.5%)	NA	NA	NA	4 (18.2%)	20 (47.6%)	0.03*
Age (yrs.)	39.5 ± 15.5	38.2 ± 16.4	41.5 ± 13.8	0.41	40.6 ± 16.4	38.9 ± 15.1	0.83
Anatomical type	*n* = 58	*n* = 37	*n* = 21	0.40	*n* = 21	*n* = 37	0.38
Diffuse	40 (69.0%)	24 (64.9%)	16 (76.2%)		13 (61.9%)	27 (73.0%)	
Localized	18 (31.0%)	13(35.1%)	5 (23.8%)		8 (38.1%)	10 (27.0%)	
Histopathological type	*n* = 28	*n* = 20	*n* = 8	0.40	*n* = 7	*n* = 21	0.97
Epithelioid	15 (53.6%)	8 (40.0%)	7 (87.8%)		4 (57.1%)	11 (52.4%)	
Sarcomatous	4 (14.3%)	4 (20.0%)	0 (0.0%)		1 (14.3%)	3 (7.1%)	
Mixed	9 (32.1%)	8 (40.0%)	1 (12.5%)		2 (28.6%)	7 (16.7%)	
Complications	*n* = 51	*n* = 31	*n* = 20		*n* = 18	*n* = 33	
Tamponade	19 (37.3%)	11 (35.5%)	8 (40.0%)	0.77	9 (50.0%)	10 (30.3%)	0.23
Constriction	14 (27.5%)	9 (29.0%)	5 (25.0%)	0.75	6 (33.3%)	8 (24.2%)	0.71
Pleural effusion	*n* = 38	*n* = 23	*n* = 15		*n* = 11	*n* = 27	0.76
Left	9 (23.7%)	7 (30.4%)	2 (13.3%)		3 (27.3%)	6 (22.2%)	
Right	3 (7.9%)	0	3 (20.0%)		0 (0%)	3 (11.1%)	
Bilateral	21 (55.3%)	13 (56.5%)	8 (53.3%)		6 (54.5%)	15 (55.6%)	
None	5 (13.2%)	3 (13.0%)	2 (13.3%)		2 (18.2%)	3 (11.1%)	

*HR* heart rate, *SBP* systolic blood pressure, *DBP* diastolic blood pressure, *NA* not applicable

*indicates *P* < 0.05 between male and female patients

### Echocardiographic characteristics

The echocardiographic sings of the study population are presented in Table 2. No difference was found between male and female patients or across time periods (all *P* > 0.05). Of all, pericardial effusion was the most frequent finding (55, 85.9%), most (43/55, 67.2%) of which was massive. Notably, PPM with no pericardial effusion was reported in > 10% of the population. Forty-three cases reported pericardiocentesis, of whom 9 (20.9%) detected malignant mesothelial cells from pericardial effusion cytological examinations. In 44 cases describing the color of pericardial effusion, 42 (95.4%) was bloody (including one case alternating with bloody and faint yellow, and another one mixed with purulent component), and the other 2 (4.5%) purely faint yellow. Twenty-six cases reported subsequent response to pericardiocentesis: 19 (73.1%) showed rapid re-accumulation in short term after pericardiocentesis, 4 (15.4%) showed temporary decrease in effusion amount but rapid progression of pericardial wall thickening and constrictive pericarditis, and the other 3 (11.5%) reported decreased amount. On the other hand, 22 cases were initially misdiagnosed as tuberculous pericarditis and received antituberculosis therapy. All these patients received pericardiocentesis and 17 cases reported the response of antituberculosis therapy: 11 (64.7%) showed rapid re-accumulation after several weeks of therapy, one of whom developed constrictive pericarditis and 6 (35.3%) showed decreased effusion and development of constrictive pericarditis.

**Table 2:** Echocardiographic findings of PPM

Parameters	All patients (*n* = 64)	Group based on sex		*P*	Group based on publishing year		
		Male (*n* = 40)	Female (*n* = 24)		1981 to 2000 (*n* = 22)	2001 to 2015 (*n* = 42)	*P*
Pericardial effusion (*n* = 64)		*n* = 40	*n* = 24	0.86	*n* = 22	*n* = 42	0.48
Mild	6 (9.4%)	3 (7.5%)	3 (12.5%)		0 (0%)	6 (14.3%)	
Moderate	6 (9.4%)	6 (15.0%)	0 (0%)		1 (4.5%)	5 (11.9%)	
Massive	43 (67.2%)	26 (65.0%)	17 (70.8%)		17 (77.3%)	26 (61.9%)	
None	9 (14.1%)	5 (12.5%)	4 (16.7%)		4 (18.2%)	5 (11.9%)	
Echo-Pericardial thickening (*n* = 64)	12 (18.8%)	7 (17.5%)	5 (20.8%)	0.83	4 (18.2%)	8 (19.0%)	0.61
Echo- Pericardial mass (*n* = 64)	21 (32.8%)	13 (32.5%)	8 (33.3%)	0.94	4 (18.2%)	17 (40.5%)	0.09
Comparable Pericardial wall thickening (*n* = 52)^a^		*n* = 32	*n* = 20		*n* = 20	*n* = 32	
Frequency on echo	9 (17.3%)	5 (15.6%)	4 (20.0%)	0.94	3 (15.0%)	6 (18.8%)	0.73
Frequency on other exams	33 (63.5%)	21 (65.6%)	12 (60.0%)	0.72	12 (60%)	21 (65.6%)	0.77
Comparable pericardial mass (*n* = 55)^a^		*n* = 35	*n* = 20		*n* = 20	*n* = 35	
Frequency on echo	20 (36.4%)	13(37.1%)	7 (35.0%)	0.87	4 (20.0%)	16 (38.1%)	0.06
Frequency on other exams	32 (58.2%)	19 (54.3%)	13 (65.0%)	0.57	10 (50.0%)	22 (62.8%)	0.40

^a^Referring to the patients with both echocardiography and at least one another examination including cardiac CT, CMR, PET-CT, exploratory thoracotomy and autopsy

Pericardial wall thickening was reported in 12 (18.8%) and pericardial cavity mass was found in 21 (32.8%) of all 64 patients, far less than the rate of pericardial effusion (85.9%). Particularly, we focused on the cases having echocardiogram and ≥ 1 another examination to assess the efficacy of echocardiography in identifying pericardial wall thickening and pericardial cavity masses in PPM (Table 2). In 52 cases that pericardial thickening could be determined by both echo and other examinations, the frequency of pericardial wall thickening was 63.5% (33/52) on non-echo exams, contrasting with an echocardiographic positive rate of only 17.3% (9/52, *P* = 0.000).

In 55 cases that pericardial mass could be identified by non-echo examinations (3 cases more than the counting of pericardial thickening were confirmed by histopathological description), 32 (58.2%) recorded pericardial masses, while echocardiography was positive in only 20 (36.4%) of the 55 patients (*P* = 0.000). Notably, 5 cases were detected on repeat echocardiography after pericardiocentesis when the amount of effusion decreased. In 43 cases reporting the location of masses: 5 (11.6%) had masses at > 3 or multiple sites, 12 (27.9%) located around the left ventricle, 8 (18.6%) around the right ventricle, 3 (6.9%) around the left atrium and 4 (9.3%) around (including one extending into) the right atrium, and the remaining 11 (25.6%) around the cardiac base, surrounding or compressing the major vessels, of which two reported thrombosis in the superior vena cava. Furthermore, 17 cases reported the echodensity of masses: 10 (58.8%) were solid, 4 (23.5%) were of mixed echodensity, and 3 (17.6%) were echolucent. The coexistence of pericardial effusion, thickening and mass was also assessed. Based on the echocardiographic data, among 55 cases of pericardial effusion, only 2 (3.6%) cases had both pericardial thickening and mass.

Agreement analysis showed poor agreement of echocardiography and non-echo examinations in identifying pericardial wall thickening (Kappa = 0.21, *P* = 0.02) and pericardial mass (Kappa = 0.58, *P* = 0.000). Sex stratified analysis also revealed a lower detectable rate of echocardiography in identifying pericardial thickening and pericardial mass (Table 2).

Ventricular wall hypokinesis was observed in 3 (4.7%) of all patients on echocardiography. While in 44 patients providing detailed description on thoracotomy (*n* = 42) or autopsy (*n* = 2), 20 (45.4%) found adhesion of malignant mesothelial tissue to ventricular wall, among whom only one (5.0%) reported wall motion hypokinesis by echocardiography.

Furthermore, we analyzed the 58 patients whose anatomic classifications could be determined. No statistically significant difference in clinical measures or occurrence of pericardial effusion was found between the diffuse and localized groups (Table 3). The detection rate of pericardial thickening in patients with diffuse type was significantly higher than that of the localized type (Table 3). The occurrence of pericardial mass was higher in patients with localized type than the diffuse type PPM on both echocardiography and non-echo examinations (*P* < 0.05).

**Table 3:** Echocardiographic features of PPM patients with two anatomic classifications

Parameter	Diffuse type (*n* = 40)	Localized type (*n* = 18)	*P* value
Age (years)	39.3 ± 16.4	38.6 ± 14.0	0.88
Gender (male, %)	24 (60%)	13 (72.2%)	0.39
Pericardial effusion Size (*n* = 58)			
Mild	2 (5.0%)	3 (16.7%)	
Moderate	6 (15.0%)	0 (0%)	
Massive	26 (65.0%)	11 (61.1%)	
None	6 (15.0%)	4 (22.2%)	0.58
Pericardial wall thickening (*n* = 48)^a^	*n* = 33	*n* = 15	
Frequency on echo	9 (27.3%)	0 (0%)	0.04
Frequency on other exams	31 (93.9%)	2 (13.3%)	0.00
Pericardial cavity mass (*n* = 51)^a^	*n* = 34	*n* = 17	
Frequency on echo	8 (23.5%)	9 (52.9%)	0.04
Frequency on other exams	11 (32.4%)	17 (100%)	0.00
In-hospital mortality (*n* = 36)	*n* = 24	*n* = 12	
	5 (20.8%)	1 (8.3%)	0.64

^a^including cases with at least one another cardiac examinations

### Survival analysis

Thirty-nine cases reported in-hospital mortality, of whom 6 (15.4%) died. Logistics analysis of parameters associated with in-hospital mortality is presented in Table 4. Potential clinical confounders (age, gender, cardiac tamponade, constrictive pericarditis and treatment with surgery) and echocardiographic parameters were included into analysis, but no predictive parameter was found (all *P* > 0.05). The analysis did not include histopathological type (*n* = 28) as all the 16 cases reporting histopathological type and outcome survived the index hospitalization.

**Table 4:** Binary Logistics analysis for predictors of in-hospital mortality

Variables	OR (95% CI)	*P* value
Age	1.00 (0.96–1.08)	0.52
Gender	1.33 (0.21–8.58)	0.76
Anatomical type	0.34 (0.04–3.35)	0.36
Echocardiographic signs		
Pericardial effusion	2.27 (0.46–11.3)	0.31
Pericardial thickening	1.22 (0.11–13.97)	0.87
Pericardial mass	2.75 (0.33–22.92)	0.35
Signs confirmed by other examinations^a^		
Pericardial wall thickening	1.67 (0.15–18.2)	0.67
Pericardial cavity mass	0.67 (0.08–4.08)	0.70
Cardiac tamponade	0.71 (0.11–4.51)	0.71
Constrictive pericarditis	1.11 (0.17–7.2)	0.91
Surgery	0.57 (0.08–4.08)	0.58

^a^including CT, CMR, PET-CT and invasive procedures (thoracotomy and autopsy)

## Discussion

In the present study, the echocardiographic characteristics of patients with histologically diagnosed PPM in China mainland in the past 35 years were systematically reviewed. According to our observations, pericardial effusion was the most common echocardiographic finding of PPM, followed by masses in the pericardial sac and pericardial thickening. The coexistence of these findings, however, is uncommon. No sex difference of prognostic echocardiographic parameters was identified.

PPM is an extremely rare but highly aggressive primary pericardial malignant tumor [1, 3–6]. Most relevant literature thus far has been case reports, resulting in poor knowledge about its echocardiographic findings. We enrolled only cases having histopathological diagnoses, as the definitive diagnosis of PPM relies on histopathological examinations [7]. This study also showed that the positive rate of pericardial effusion is low (20.9%), similar to previous report (24%) [8].

The analyzed patients showed a male/female ratio of 1.7:1, similar to a recent study by Mensi et al., who reported a male/female ratio of 1.79 in 4442 patients with malignant mesothelioma [3]. We also found that the middle-aged group was mostly affected (85.9%) with a mean age of 39.5 years. PPM of diffuse (69.0%) and epithelioid type (53.6%) was more commonly seen. Our study confirmed that the hemodynamic complications of cardiac tamponade and constrictive pericarditis in PPM were quite common (37.3% and 27.5% respectively), which have been reported frequently [9–11]. It has been suggested that the presence of cardiac tamponade increases the likelihood of malignancy [11]. This may be associated with the diffuse mesothelial cell proliferations and myocardial infiltration which may decrease the relaxation and compliance of left ventricle.

### Pericardial effusion

This study showed that the most common echocardiographic sign of PPM, regardless of gender or morphological type, is pericardial effusion (85.9%), which is often massive (67.2%), bloody (95.4%) and associated with hemodynamic instability. When determining the etiology of pericardial effusion, it is crucial to exclude other more commonly seen causes, such as metastatic malignant effusion or inflammatory pericardial diseases [12, 13], considering the rarity of PPM. Timely pericardiocentesis helps to stabilize hemodynamics, and yet we found that rapid re-accumulation of massive effusion after days or weeks of pericardiocentesis is quite common (73.1%). This phenomenon may suggest the malignancy of etiology, helpful for differentiating with tuberculosis [14]. Interestingly, there is also a number of patients (15.4%) who showed temporarily decreased effusion but rapid progression into pericardial thickening and development of constrictive pericarditis. This may be caused by the mechanical injury with pericardiocentesis, which might stimulate malignant mesothelial cells proliferation. However, whether this response to pericardiocentesis is specific to PPM could not be determined from this retrospective study.

It should be noted that PPM with no or minimal pericardial effusion, and significant pericardial thickening, constriction or even occlusion of pericardial cavity was not uncommon (> 10%). Lee et al [10] reported a similar case in a 59-year-old woman of epitheliod PPM, who had marked pericardial thickening but no fluid on echocardiography. This may be associated with the multi-differentiating potential of mesothelial cells, different percentiles of the fibrous component takes or tumor invasiveness of the vessels.

### Pericardial mass

According to our data, the pericardial mass may develop in any place around the visceral or parietal pericardium. Notably, masses around the cardiac base (25.6%) tend to be large and aggressive for adjacent structures. In the analyzed patients, two cases developed superior vena caval thrombosis [14]. Nguyen et al [5] observed that patients with mesothelioma are susceptible to thromboembolic events (27.7%), probably correlated with excessive release of procoagulant factors. Also, we found that the echocardiographic detectable rate of pericardial mass is less than that of other examinations performed in the same patients (36.4% vs.58.2%, *P* = 0.000). In consideration of the indicative value of detecting pericardial mass in narrowing down differential diagnoses, this finding supports the routine screening for pericardial mass when assessing pericardial fluid.

### Pericardial thickening

Our data showed that the majority (93.9%) of diffuse type PPM patients have diffused pericardial thickening, more than that of the localized type (13.3%). This may result from the difference in mesothelial cell growth and proliferation velocity between the two phenotypes. The frequency of echo identified pericardial thickening is no more than 20%, far less than the rate (63.5%) confirmed by other examinations, supporting the superiority of cardiac CT and CMR in determining the thickening or tissue characteristics of pericardial walls [15]. Jiang et al. [16] reported a patient with sarcomatoid PPM whose echocardiogram indicated only pericardial mass and effusion, while both cardiac CT and PET scan confirmed a thickened pericardium. The suboptimal pericardial wall visualization of echocardiogram, difficulty to discern between pericardial thickening and epicardial fat or masses of other nature is responsible for its low detection rate, particularly under emergency bedside condition when more attention was given to the effusion or ventricular function assessment.

### Myocardial involvement

It is noteworthy that myocardial infiltration confirmed by open heart surgery is as high as 45.4% in the present study, in contrast with only 5.0% of wall motion hypokinesis on echocardiography. This finding not only indicates the aggressive nature of PPM but implies the insensitivity of conventional echocardiographic parameters to detect myocardial lesions in PPM. The use of speckle tracking imaging to assess cardiac strain has been confirmed to be sensitive of detecting subclinical myocardial impairment by a large body of evidence, and might be helpful to detect early subtle myocardial lesions in PPM [17]. Severe case with myocardial necrosis was reported in one 77-year-old male, manifesting as severe dyspnea, ST segment elevation, increased cardiac troponin level and hypokinetic ventricular wall motion in the patient cohort, similar to a 75-year-old woman with suspected ST-elevation myocardial infarction reported by Barroso et al. [18].

It is difficult to define the “typical” of “classical” signs of PPM from this study. The coexistence of massive pericardial effusion, pericardial mass, thickening and signs of cardiac tamponade seems to be indicative of malignant etiology but is uncommon and nonspecific [19]. PPM should be considered after excluding the much more frequent metastatic or benign pericardial tumors. The present study found an in-hospital mortality of 15.4% of PPM, but no prognostic clinical or echocardiographic parameters could be determined, possibly limited by the relatively small population and end-point events.

### Study limitations

Due to the extreme rarity of PPM incidence, we can only perform a retrospective analysis and included a limited number of patients. Thus, the association between asbestos exposure and PPM, which has been controversial [8, 20], cannot be determined. Besides, Selection bias that is inherent to retrospective study and the fact that complete information was not available in each and all patient, represent a major limitation of the study. However, for diseases of rare incidence, retrospective review of cases from multiple institutions or across a wide time range is valuable for accumulating knowledge about the index disease [21]. Consequently, we enrolled only histopathologically diagnosed cases and cases with individual clinical and echocardiographic information as these may have relatively complete data. However, only inclusion of patients with echocardiographic data might limit the extrapolation of our results. Further prospective nation-wide registry study of PPM is required. It is also noteworthy that the echocardiographic findings of PPM described in our study are all nonspecific. The definitive diagnosis of PPM remains to be histopathological tests.

## Conclusion

Our study revealed the echocardiographic features of PPM and showed that massive pericardial effusion is the most common echocardiographic sign, although the signs are generally nonspecific and had no gender-specific difference or association with in-hospital mortality. The detecting ability of echocardiogram for pericardial wall lesions and the yield of pericardiocentesis are not perfect, indicating the necessity for timely histopathological examinations to confirm diagnosis in suspected cases.

## Abbreviations

CMR: magnetic resonance
CT: computed tomography
PET: Positron Emission Tomography
PPM: Primary malignant pericardial mesothelioma
